# Age-related differences in management and outcomes in hospitalized healthy and well-functioning bacteremic pneumococcal pneumonia patients: a cohort study

**DOI:** 10.1186/s12877-017-0518-0

**Published:** 2017-06-20

**Authors:** Luis A. Ruiz, Pedro P. España, Ainhoa Gómez, Amaia Bilbao, Carmen Jaca, Amaia Arámburu, Alberto Capelastegui, Marcos I. Restrepo, Rafael Zalacain

**Affiliations:** 1 Pneumology Service, Hospital Universitario Cruces, E-48903 Barakaldo, Bizkaia Spain; 2 Pneumology Service, Hospital Galdakao-Usansolo, Galdakao, Bizkaia Spain; 3 Research Unit, Hospital Universitario Basurto – Research Network on Health Services for Chronic Diseases (REDISSEC), Bilbao, Bizkaia Spain; 4 Division Pulmonary/Critical Care Medicine, South Texas Veterans Health Care System and University of Texas health Science Center, San Antonio, TX USA

**Keywords:** Bacteremic pneumococcal pneumonia, Community-acquired pneumonia, Pneumonia in older people

## Abstract

**Background:**

Limited data are available regarding fit and healthy patients with pneumonia at different ages. We evaluated the association of age with clinical presentation, serotype and outcomes among healthy and well-functioning patients hospitalized for bacteremic pneumococcal community–acquired pneumonia.

**Methods:**

We performed a prospective cohort study of consecutive healthy and well-functioning patients hospitalized for this type of pneumonia. Patients were stratified into younger (18 to 64 years) and older (≥65 years) groups.

**Results:**

During the study period, 399 consecutive patients were hospitalized with bacteremic pneumococcal pneumonia. We included 203 (50.8%) patients who were healthy and well-functioning patients, of whom 71 (35%) were classified as older. No differences were found in antibiotic treatment, treatment failure rate, antibiotic resistance, or serotype, except for serotype 7F that was less common in older patients. In the adjusted multivariate analysis, the older patients had higher 30-day mortality (OR 6.83; 95% CI 1.22–38.22; *P* = 0.028), but were less likely to be admitted to the ICU (OR 0.14; 95% CI 0.05–0.39; *P <* 0.001) and had shorter hospital stays (OR 0.71; 95% CI 0.54–0.94; *P* = 0.017).

**Conclusions:**

Healthy and well-functioning older patients have higher mortality than younger patients, but nevertheless, ICU admission was less likely and hospital stays were shorter. These results suggest that the aging process is a determinant of mortality, beyond the functional status of patients with bacteremic pneumococcal pneumonia.

## Background

The incidence of pneumonia and associated mortality are higher in older than younger people. Pneumonia is the third most frequent cause of hospitalization in patients aged 65 years or over [1], streptococcus pneumoniae being the main pathogen isolated. Bacteremic pneumococcal pneumonia constitutes a severe subgroup with its own features.

Many previous studies have found that the mortality risk among older patients with pneumonia depends on the severity of the lung infection, and adequacy of the response to the infection and other host factors including comorbidities and low functional status [2, 3]. Ageing is among the most important known risk factors for most chronic diseases.

Older patients with pneumonia tend to have multiple comorbid chronic conditions leading to loss of functional independence and an inadequate response to the infectious process. The role of age in mortality prediction is controversial due to interactions between age and comorbidities. Further, pneumonia itself can trigger acute mobility impairment and delirium in this population. All of these factors are markers of frailty and increase the likelihood of poor outcomes [4–6]. Frailty refers to an individual’s increased susceptibility towards adverse clinical events, and it is becoming recognized that frailty represents a dynamic geriatric syndrome distinct from, but overlapping with, comorbidities and disability [7–9]. On the other hand, improvements in social and health conditions together with a rise in life expectancy have resulted in an increase in the number of “healthy” and well-functioning older people. There is limited information, however, regarding process of care and outcomes of common medical conditions requiring hospital admission in this subgroup of fit and healthy older patients.

Aging is characterized by progressive tissue degeneration leading to a negative effect on the structure and function of vital organs even in the absence of comorbid illness [10]. For this reason, we hypothesized that the survival of healthy older patients would be poorer than that in younger patients. To test this hypothesis, our aim was to assess the association of age with clinical presentation, serotype, process of care and outcomes among healthy and well-fitted older patients hospitalized for bacteremic pneumococcal community–acquired pneumonia.

## Methods

### Study design and population

This was a prospective observational study of consecutive patients hospitalized for bacteremic pneumococcal pneumonia (positive blood culture taken within 24 h after admission) in two tertiary medical centers. The study was conducted between January 2002 and January 2010. The ethics committees of Cruces and Galdakao Hospitals approved the study.

The healthy and well-functioning patients´ state condition was assessed using the Clinical Frailty Scale (CFS) [11]. This tool provides a quick and easy estimation based on clinical judgment and quantifies frailty on a scale ranging from 1 (very fit) to 9 (terminally ill). Each patient was assigned a score on the CFS by two seniors researchers using data extracted from Cruces/Galdakao prospective pneumonia registry. For the purpose of the study, we only included patients who were considered independent in activities of daily living and had no medical comorbidities (categories 1, “very fit”, and 2, “well”, of the CFS). These patients were stratified into two groups according to their age: 1) younger adults (18–64 years); and 2) older adults (≥ 65 years).

### Study variables

Since 2000, there has been an ongoing prospective and standardized registry of all patients hospitalized for pneumonia in our two hospitals. This registry includes multiple variables characterizing patients and their pneumonia. For eligible patients, we assessed data on socio-demographic characteristics (including ability to carry out basic activities of daily living, self-care activities and regular physical activity), medical comorbidities, influenza and pneumococcal vaccination status, vital signs, results of routine laboratory tests, including the pneumococcal urinary antigen test, and radiological findings on admission. Patients were empirically treated in accordance with the National Guidelines of the Spanish Society of Pulmonology [SEPAR] [12] at the discretion of the attending doctor. The severity of patients’ clinical condition was assessed on admission using the *CURB-65 score* [13]. All survivors were followed up to 30 days after discharge or until complete radiological resolution.

Two consecutive blood cultures were taken for all patients within 24 h after hospital admission. Tests were conducted to assess the susceptibility of *Streptococcus pneumoniae* to the following antibiotics: penicillin, ceftriaxone, erythromycin and levofloxacin. Pneumococcal serotypes were assessed and grouped according to the associated risk of mortality into the following categories: 1) high risk, serotypes 3, 6A, 6B, 9 N, 19F, 19A, and 23F; 2) intermediate risk, serotypes 9 V, 12F, 14, and 22F; and 3) low risk, serotypes 1, 7F, 8, 4 and 5 [14].

### Clinical outcomes

To assess the treatment, the following variables were studied: 1) appropriateness of the empirical antibiotic used (according to the recommendations of the Spanish Society of Pulmonology [SEPAR]) [12]; 2) and 3) whether antibiotic treatments were started within 4 or 8 h after arrival at the emergency department, respectively; and 4) the class of antibiotics administered.

Clinical in-hospital course and outcome measures included: 1) admission to the intensive care unit (ICU); 2) use of invasive mechanical ventilation; 3) septic shock; 4) in-hospital, early (≤ 48 h) [15] and 30-day mortality; 5) treatment failure; 6) length of hospital stay; and 7) 30-day readmission.

### Definitions

Pneumonia was defined as the presence of new pulmonary infiltrate on the chest X-ray together with signs and symptoms suggestive of lower respiratory tract infection. Septic shock was defined as a systolic blood pressure of less than 90 mmHg and a need for vasopressor drugs for at least 4 h, after fluid therapy [16]. Treatment was considered to have failed when patients’ clinical condition worsened during their hospital stay with: hemodynamic instability; appearance or worsening of respiratory failure; a need for mechanical ventilation; progression of the pneumonia, as indicated by radiological findings or the appearance of a new focus of infection; or persistence or reappearance of fever, if a change of treatment was required [17]. The diagnosis of altered mental status was based on observation that the patient’s mental state was not normal and that this was a new phenomenon. Comorbidities including the follow conditions: chronic respiratory disease, diabetes mellitus, chronic cardiovascular disease, neurologic disease, liver disease and chronic renal disease.

### Statistical analysis

Descriptive analysis was undertaken, using frequencies and percentages, means and standard deviations (SDs) or medians and interquartile ranges (IQRs). Patient characteristics were compared between the groups (younger vs older patients), as were variables related to treatment, serotypes, in-hospital course and outcomes. Comparisons were performed with chi-square or Fisher’s exact tests for qualitative variables, and with t tests or non-parametric Wilcoxon tests for quantitative variables.

Univariate logistic regression models were used to compare in-hospital course and clinical outcomes between the groups. Then, multivariate logistic regression models were built adjusting for CURB score (as a continuous variable), variables with *p* < 0.05 and other variables considered clinically relevant in the univariate analysis as potential independent variables. The results are reported as odds ratios (ORs) and 95% confidence intervals (CIs), considering the younger patients (age < 65 years) as the reference group. For comparing length of stay, a general linear model was used, and due to their skewed distribution, these data were log-transformed. Hence, the results are given as the exponential of the estimated beta parameter, indicating how many times longer the mean stay of older patients was than that of younger patients.

A *P* value <0.05 was considered statistically significant. All the statistical analysis was performed using the SAS software for Windows version 9.2 (SAS Institute, Cary, NC).

## Results

During the study period, a total of 4978 consecutive patients diagnosed with pneumonia were hospitalized in our two hospitals, including 399 with bacteremic pneumococcal pneumonia. Of these, 203 (50.8%) were healthy and well-functioning, 132 patients being <65 years and 71 being 65 years old or older.

Table 1 summarizes the baseline characteristics of all patients stratified by age. Younger patients were more often smokers and heavy drinkers, and were more likely to have higher heart rate and hypotension, while more of the older patients had altered mental status at admission. In addition, the older patients were more likely to have severe hypoxemia and elevated blood urea nitrogen levels. No statistically significant differences were observed in urinary antigen test or radiological imaging results. The older patients were more frequently classified in the higher risk classes of the *CURB-65 score* (3 to 5) (*P* < 0.001). None of the eligible population for this study had previously been in long-term care facilities.

**Table 1:** Demographic and clinical characteristics at admission

Characteristics	Age < 65 years (*N* = 132)	Age ≥ 65 years (*N* = 71)	*P* value
Demographics			
Male sex	92 (69.7)	36 (50.7)	0.007
Age (years), mean (SD)	43.67 (11.7)	78.27 (8)	<0.001
Vaccination status			
Influenza vaccine	3 (2.3)	23 (41)	<0.001
Pneumococcal vaccination	0 (0)	1 (1.6)	0.322
Current tobacco use	63 (56.2)	2 (4.8)	<0.001
Heavy drinker (> 80 mg alcohol/day)	29 (22.3)	6 (8.7)	0.016
Clinical characteristics at admission			
Body temperature (°C), mean (SD)	38.05 (1.1)	37.82 (1.1)	0.239
Respiratory rate, mean (SD)	22.50 (6.8)	25.06 (6.7)	0.004
Heart rate, mean (SD)	108.43 (21)	97.70 (17.6)	<0.001
Altered mental status	3 (2.2)	12 (16.9)	<0.001
Systolic blood pressure < 90 mmHg	17 (12.8)	1 (1.4)	0.006
Laboratory and radiological findings			
BUN >30 mg/dL	36 (27.2)	38 (53.5)	<0.001
PaO_2_ < 60 mmHg	39 (29.5)	47 (66.2)	<0.001
CRP (mg/dL), mean (SD)	35.23 (18.3)	28.53 (18.4)	0.191
Multilobar pneumonia	53 (40.1)	22 (31.4)	0.222
Pleural effusion	29 (21.9)	8 (11.2)	0.059
Urinary antigen positive	87 (71.3)	51 (83.6)	0.068
Antibiotic resistance			
Penicillin/amoxicillin	1 (0.7)	2 (2.9)	0.267
Ceftriaxone	0 (0)	0 (0)	─
Erythromycin	12 (9.1)	7 (10.2)	0.783
Levofloxacin	0 (0)	0 (0)	─
*CURB-65* score			<0.001
0-I	96 (72.7)	4 (5.6)	
2	31 (23.4)	38 (53.5)	
3–5	5 (3.7)	29 (40.8)	

Data are given as frequency (percentage) unless otherwise stated. Percentages exclude patients with missing data

*SD* Standard deviation, *CRP* C-reactive protein, *BUN* Blood urea nitrogen

The antibiotic treatments used are reported in Table 2. Treatment duration was shorter in the older group (13.6 vs 15.7 days, *P* = 0.022). The most common single antibiotic class administered was fluoroquinolones in both groups and differences in antibiotic class and treatment failure rate were not significant. Further, no significant differences were found in the rates of resistance to antibiotics tested. Beta-lactam in combination with a macrolide was only prescribed in four patients, all of them elderly.

**Table 2:** Indicators for healthy and well-functioning hospitalized patients with pneumococcal pneumonia

Process of care	Age < 65 years (*N* = 132)	Age ≥ 65 years (*N* = 71)	*P* value
Prior antibiotic treatment	8 (6.1)	9 (12.6)	0.104
Antibiotic within 4 h	88 (71.5)	46 (69.7)	0.789
Antibiotic within 8 h	115 (93.5)	59 (89.3)	0.320
Appropriate antibiotic	96 (72.7)	51 (71.8)	0.891
Length of antimicrobial treatment, days, mean (SD)	15.71 (7.3)	13.61 (9.12)	0.022
Antibiotic treatment			0.364
Beta-lactam	21 (15.9)	17 (23.9)	
Fluorquinolones	95 (71.9)	47 (66.2)	
Others	16 (12.1)	7 (9.8)	
Combination therapy including a macrolide	0 (0)	4 (5.6)	0.017
Treatment failure	16 (12.3)	12 (17.1)	0.347

Data are given as frequency (percentage) unless otherwise stated. Percentages exclude patients with missing data

*SD* Standard deviation

Serotype analysis was performed in 165 out of the 203 patients (81.2%), and the serotype distribution was found to vary widely. The most frequent serotypes are listed in Table 3. Of them, serotype 7F was more frequently identified in younger patients. No significant differences were observed in the rest of serotypes studied, either separately or clustered by the associated risk of death.

**Table 3:** Serotype distribution by age group

Serotype	Age < 65 years (*N* = 132)	Age ≥ 65 years (*N* = 71)	*P* value
3	17 (16)	15 (25.4)	0.143
4	11 (10.3)	2 (3.3)	0.138
8	9 (8.4)	4 (6.7)	0.772
1	15 (14.1)	7 (11.8)	0.678
19A	9 (8.4)	5 (8.4)	0.997
14	5 (4.7)	3 (5.1)	1
7F	19 (17.9)	4 (6.7)	0.033
22	4 (3.7)	3 (5.1)	0.701
Clusters			
High risk (3 + 6A+ 6B + 9 N + 19F + 19A + 23F)	28 (26.4)	22 (37.2)	0.145
Intermediate risk (9 V + 12F + 14 + 22F)	8 (7.5)	5 (8.4)	1
Low risk (1 + 7F + 8 + 4 + 5)	44 (41.5)	17 (28.8)	0.105

Data are given as frequency (percentage). Percentages exclude patients with missing data

Table 4 presents the in-hospital course and clinical outcome indicators. The older patients were less likely to be admitted to the ICU. Overall, 13 out of 203 (6.4%) patients died within 30 days. Five of these (two ≥65 years old) had been admitted to the ICU. The older patients had higher in-hospital (12.6% vs 2.2%, *P* = 0.004), early (8.4% vs 0.7%, *P* = 0.008) and 30-day (14.1% vs 2.2%, *P* = 0.001) mortality. There were no significant differences in 30-day readmission rate between groups. In the adjusted multivariate analysis, the older patients had higher 30-day mortality (OR 6.83; 95% CI 1.22–38.22; *P* = 0.028), but were less likely to be admitted to the ICU (OR 0.14; 95% CI 0.05–0.39; *P <* 0.001) and had shorter hospital stays (OR 0.71; 95% CI 0.54–0.94; *P* = 0.017) than the younger patients.

**Table 4:** In- hospital and 30-day outcomes by age group

Outcome Measures				Non-adjusted analysis		Adjusted analysis^a^
	Age < 65 years (*N* = 132)	Age ≥ 65 years (*N* = 71)	*P* value	Odds ratio (95% CI)		*P* value
In-hospital mortality	3 (2.2)	9 (12.6)	0.004	6.24 (1.63–23.87)	4.22 (0.75–23.69)	0.101
Early mortality	1 (0.7)	6 (8.4)	0.008	12.09 (1.43–102.51)	4.34 (0.36–52.36)	0.248
30-day mortality	3 (2.2)	10 (14.1)	0.001	7.05 (1.87–26.54)	6.83 (1.22–38.22)	0.028
Intensive care unit	38 (28.7)	12 (16.9)	0.060	0.50 (0.24–1.04)	0.14 (0.05–0.39)	<0.001
Invasive mechanical ventilation	13 (9.8)	4 (5.6)	0.301	0.55 (0.17–1.74)	0.32 (0.08–1.32)	0.114
Septic shock	16 (13.4)	8 (12.7)	0.887	0.94 (0.38–2.33)	0.59 (0.19–1.90)	0.377
Length of hospital stay, days^b^						
Mean (SD)^c^	11.02 (17.7)	7.50 (8.2)	0.809	0.85 (0.64–1.13)	0.71 (0.54–0.94)	0.017
Median (IQR)	6 (4–10)	5 (3–8)	0.809			
30-day readmission	2 (2.1)	1 (1.7)	1	0.83 (0.07–9.40)		

Odds ratios are calculated considering the group of patients with age < 65 years old as the reference group

^a^Adjusted analysis: Odds ratio adjusted for CURB (as a continuous variable), sex, heavy drinking, PaO_2_ < 60 mmHg, appropriate antibiotic and antibiotic within 4 h

^b^Deaths are excluded

^c^For the comparison of length of hospital stay as a continuous variable, a general linear model was used, and due to the skewed distribution of length of stay, these data were log-transformed. Hence, data is given as the exponential of the estimated beta parameter, indicating how many times longer the mean stay of patients ≥65 years was than that of those <65 years old. CI, confidence interval; IQR interquartile range; SD, standard deviation

## Discussion

The results of this study show that the mortality of healthy older people hospitalized for bacteremic pneumococcal pneumonia is higher than that of younger adult patients (<65 years old) with the same characteristics, independent of the serotype, severity and the type of care provided. Despite their good baseline health and functional status, ICU admission rates are lower in patients ≥65 years old. The interest of our study lies in the type of population studied. To our knowledge, this is the first study specifically focused on the subgroup of healthy and well-functioning young and older adults with bacteremic pneumococcal pneumonia.

In developed countries, the prognosis of pneumonia in older people has changed in the last decade, mainly due to improvements in healthcare and social conditions. As a consequence, it has recently been proposed that diagnostic and therapeutic decisions should be based more on the patients’ degree of frailty than their age [18, 19]. This concept may be a better reflection of biological than chronological age. Nevertheless, frail is not synonym for having comorbidities or functional limitations; rather it is recognized as a distinct clinical syndrome characterized by a decrease in physiological reserve and resistance to stressful situations, making individuals more vulnerable to health problems [9]. There is no consensus, however, as to how frailty should be assessed. There are many definitions, the majority of which are based on complex scales and which have not gained acceptance among practicing clinicians [20]. The CSHA Clinical Frailty Scale is a simple and reproducible tool that provides a realistic and simple way to assess the reality of these patients in different medical conditions [21, 22].

The prevalence rates of chronic disease and functional impairment in adults increase proportionally with age [23]. Nonetheless, the contribution of underlying diseases to outcomes in patients with pneumonia remains somewhat controversial [24–26]. Further, a recent study has shown that serotype rather than the presence of comorbidities is the most important risk factor for the development of respiratory failure in patients with pneumococcal pneumonia [27]. Conversely, evidence on the role of functional status seems to be one of the most consistent predictors of poor clinical outcomes [28, 29].

Several studies have investigated the association between frailty and outcomes in hospital and after discharge but none of them to our knowledge have been designed to compare healthy and well-functioning patients stratified by age [21, 30, 31]. Although there are no specific studies on this population, it is generally assumed that there are no significant differences between the management of older and younger patients with the same characteristics [18]. In this study, however, we found that older patients have higher 30-day mortality. Other authors have reported a poorer prognosis for pneumonia in elderly patients without comorbidities but none of these studies have been adjusted for severity [32, 33].

The reason for this poorer outcome is not clear. Despite the fact that we have previously reported that mortality in elderly patients with bacteremic pneumococcal pneumonia is associated with age and the severity of the infectious condition itself, the role of age as a prognostic factor is controversial [34, 35]. From a theoretical point of view, a healthy non-frail patient might represent a good model for studying the effect of ageing itself on the management and prognosis of multiple diseases. In our study, given the lack of significant differences in the class of antibiotics provided and virulence of the serotype involved, age seems likely to be relevant to prognosis. In fact, a causative role of immunosenescence in the outcome of older patients seems highly plausible [36]. Aging is associated with changes in immune response impairment of alveolar macrophage function and increases in cellular apoptosis during sepsis, leading to a greater severity of infection [37]. Nevertheless, other authors have reported that age itself did not have any impact on mortality in patients with one or no comorbid conditions, except for those aged 80 years and older [38]. In our study, we cannot exclude in a subgroup of patients a pre-frail stage revealing a vulnerable state of relatively low physiological reserve to respond adequately to any acute clinical deterioration**.** Such a state may identify a subset of patients who are at high risk of progressing to frailty or reverse to non-frail under external stressors [9].

On the other hand, it is recognized that age itself is an important limiting factor for ICU admission, independent of baseline status and severity of illness [39–42]. However, to our knowledge no previous studies have focused on older patients who were fit and “healthy”. Given the progressive aging of the population, we should consider changing ICU admission criteria to take into account biological age, more than chronological age.

Our study has some limitations: 1) We have not used any functional assessment scale, and hence, we cannot completely rule out a certain degree of functional limitation in some patients. The effect of any misclassification in this study would be limited, because according to some authors self-reported measures of mobility limitation are well correlated with other objective scales [43, 44]. 2) Due to the characteristics of our registry, we may not have adequately identified the subgroup of “vulnerable” patients (CFS = 3, 4), namely, those who are not dependent but do have some limitations and complain of being “slowed up” or tired [11, 30]. Although such patients should not be considered frail, we cannot rule out a pre-frail state having contributed to the poor outcome in some patients. Further, we have not assessed the role of concomitant medications that could have influenced the outcome of these patients [45]. 3) There was a low rate of inpatient death, and this is reflected in wide CIs. This is attributable to the type of population eligible for this study and the marked reduction in mortality among pneumonia patients in recent years. 4) The observational study design could have introduced bias. In particular, decisions on the process of care were left to the discretion of the managing physicians. The effect of this potential source of heterogeneity may be limited by the high degree of reliability and prospective collection of data, together with the adjustment for potential confounding variables. Given this and to avoid possible age bias, the multivariate logistic regression models adjusted for CURB score (excluding age). In our opinion, this design represents a realistic approach to investigate the real-world care of patients with pneumonia. Despite these limitations, we believe that this study has produced important findings that should be considered in the management of older patients.

## Conclusions

In this study, we have described the observation that bacteremic pneumococcal pneumonia in healthy and well-functioning older patients behaves as a clinical entity distinct from that in younger patients, and notably, outcomes are poorer in the older age group. These differences in survival do not seem to be explained by process of care or serotype. Future multicentre studies are required to confirm these results. In the meantime, we suggest that biological age should be more routinely assessed to guide clinical decision making in older patients in general and, in particular, to help clinicians identify older patients with pneumonia who might benefit from ICU admission.
